# Supplementary material for: Structures of human dynein in complex with the lissencephaly 1 protein, LIS1
Source: eLife. 2023 Jan 24;12:e84302. doi: 10.7554/eLife.84302 (PMC9889085; doi:10.7554/eLife.84302)
Supplement: Supplementary file 1. — CryoEM data collection parameters, reconstruction information and model refinement statistics for the structures of human dynein bound to one and two LIS1 β-propellers. [file elife-84302-supp1.docx]

**Supplementary File 1: Cryo-EM data information and model validation.**

|  |  | | |  |
| --- | --- | --- | --- | --- |
| **Models** | **Human Dynein - Lis1** | | | **Human Dynein - (Lis1)_2_** |
| PDB  EMDB  EMPIAR | 8DYV  EMD-27783  EMPIAR-11373 | | | 8DYU  EMD-27782  EMPIAR-11373 |
| **Data collection and processing** | | |  | |
| Microscope  Camera  Magnfication |  | Talos Arctica  Gatan K2 Summit  36 000 | | |
| Voltage (kV) |  | 200 | | |
| Electron exposure (e–/Å^2^) |  | 58 | | |
| Defocus range (μm) |  | 0.3 – 3.7 | | |
| Pixel size (Å)  Number of datasets (no.) |  | 1.16  3 | | |
| **Reconstruction** |  | | |  |
| Symmetry imposed | C1 | | | C1 |
| Initial particles(no.) | 996 929 | | | 996 929 |
| Final particles (no.) | 24 217 | | | 37 288 |
| Micrographs collected (no.)  Micrographs final (no.)  Map resolution (Å) (0.143 FSC threshold) | 3763  2892  4.0 | | | 3763  2892  4.1 |
|  |  | | |  |
| **Model Refinement and Validation** | | |  |  |
| Initial model used | PDB 5NUG; AlphaFold P43034 | | | Human Dynein-Lis1 |
| Map-to-model resolution  (0.5 FSC threshold) (Å) | 4.3 | | | 4.4 |
| Map sharpening *B* factor (Å^2^)  Model to Map Fit  CC mask | 50.3  0.70 | | | 65.4  0.68 |
| *Model composition*  Non-hydrogen atoms  Protein residues  Ligands | 23 053  2 854  4 | | | 24610  3174  4 |
| *B* factors (Å^2^)  Protein  Ligand | 111  89 | | | 276  49 |
| *R.m.s. deviations*  Bond lengths (Å)  Bond angles (°) | 0.006  1.200 | | | 0.005  0.868 |
| *Validation*  MolProbity score  Clashscore  Poor rotamers (%) | 1.56  5.74  0.20 | | | 1.39  4.12  0.23 |
| *Ramachandran*  Favored (%)  Allowed (%)  Disallowed (%) | 96.33  3.63  0.04 | | | 96.83  3.14  0.03 |
