# Supplementary material for: Structures of human dynein in complex with the lissencephaly 1 protein, LIS1
Source: eLife. 2023 Jan 24;12:e84302. doi: 10.7554/eLife.84302 (PMC9889085; doi:10.7554/eLife.84302)
Supplement: Supplementary file 2. — This table lists the mutations in dynein and LIS1 that are associated with different disease and are shown in Video 2, along with the references reporting them. Video 2 shows (and this Table lists) only residues where we determined that the reported mutation(s) do not have an obvious destabilizing effect based on an inspection of the structure. The color coding in the table corresponds to that used in Video 2. [file elife-84302-supp2.docx]

**Supplementary File 2: Disease mutations in dynein and LIS1 shown in Video 2.**

|  | **Protein** | **Mutation** | **Reference** |
| --- | --- | --- | --- |
| Lissencephaly | LIS1 | Arg20Ser | (Uyanik et al., 2007) |
|  |  | Ala210Pro | (González-Morón et al., 2017) |
|  |  | His277Pro | (Torres et al., 2004) |
|  |  | Arg342Pro | (Jamuar et al., 2014) |
|  |  | Asp387Asn | (Saillour et al., 2009) |
|  |  | His389Tyr | (Mineyko et al., 2010) |
| Charcot-Marie-Tooth | Dynein | Val1750Met | (Amabile et al., 2020) |
|  |  | Gln1860Lys | (Amabile et al., 2020) |
|  |  | Arg1887His | (Amabile et al., 2020) |
|  |  | Tyr2641Cys | (Antoniadi et al., 2015) |
|  |  | Glu2814Asp | (Amabile et al., 2020) |
|  |  | Val3065Met | (Amabile et al., 2020) |
|  |  | Arg3989Cys | (Amabile et al., 2020) |
|  |  | Thr4450Met | (Amabile et al., 2020) |
|  |  | Leu4577Pro | (Amabile et al., 2020) |
| Spinal Muscular Atrophy | Dynein | Glu2616Lys | (Scoto et al., 2015) |
|  |  | Glu3048Lys | (Fiorillo et al., 2014) |
| Autism Spectrum Disorders | Dynein | Ala2233Val | (Amabile et al., 2020) |
|  |  | Asp2573Gly | (Amabile et al., 2020) |
|  |  | Glu3025Lys | (C Yuen et al., 2017) |
|  |  | Gly4072Ser | (Iossifov et al., 2014) |
|  |  | Asn4266Ile | (Amabile et al., 2020) |
| Malformations of cortical development | Dynein | Arg1962Cys | (Poirier et al., 2013) |
| Intellectual disability |  | Arg1962Leu | (Zhu et al., 2015) |
|  |  | Arg2025Gln | (Meng et al., 2017) |
|  |  | Arg2332Cys | (Zhu et al., 2015) |
|  |  | Asp2573Gly | (Amabile et al., 2020) |
|  |  | Arg2720Lys | (Zillhardt et al., 2016) |
|  |  | Glu3048Lys | (C Yuen et al., 2017) |
|  |  | Ala3084Thr | (Trujillano et al., 2017) |
|  |  | Arg3474Trp | (Amabile et al., 2020) |
|  |  | Arg3525Cys | (Bowling et al., 2017) |
|  |  | Gly3630Ser | (Amabile et al., 2020) |
|  |  | Gly3658Glu | (Al-Shamsi et al., 2016) |
|  |  | Arg3728Pro | (Amabile et al., 2020) |
|  |  | Glu3771Lys | (Amabile et al., 2020) |
|  |  | His3822Pro | (Vissers et al., 2010) |
|  |  | Pro3942Ser | (Hayashi et al., 2017) |
|  |  | Val3951Ala | (Laquerriere et al., 2017) |

**References**

Al-Shamsi A, Hertecant JL, Souid A-K, Al-Jasmi FA. 2016. Whole exome sequencing diagnosis of inborn errors of metabolism and other disorders in United Arab Emirates. *Orphanet Journal of Rare Diseases* **11**:94. doi:10.1186/s13023-016-0474-3

Amabile S, Jeffries L, McGrath JM, Ji W, Spencer-Manzon M, Zhang H, Lakhani SA. 2020. DYNC1H1-related disorders: A description of four new unrelated patients and a comprehensive review of previously reported variants. *Am J Med Genet A* **182**:2049–2057. doi:10.1002/ajmg.a.61729

Antoniadi T, Buxton C, Dennis G, Forrester N, Smith D, Lunt P, Burton-Jones S. 2015. Application of targeted multi-gene panel testing for the diagnosis of inherited peripheral neuropathy provides a high diagnostic yield with unexpected phenotype-genotype variability. *BMC Med Genet* **16**:84. doi:10.1186/s12881-015-0224-8

Bowling KM, Thompson ML, Amaral MD, Finnila CR, Hiatt SM, Engel KL, Cochran JN, Brothers KB, East KM, Gray DE, Kelley WV, Lamb NE, Lose EJ, Rich CA, Simmons S, Whittle JS, Weaver BT, Nesmith AS, Myers RM, Barsh GS, Bebin EM, Cooper GM. 2017. Genomic diagnosis for children with intellectual disability and/or developmental delay. *Genome Med* **9**:43. doi:10.1186/s13073-017-0433-1

C Yuen RK, Merico D, Bookman M, L Howe J, Thiruvahindrapuram B, Patel RV, Whitney J, Deflaux N, Bingham J, Wang Z, Pellecchia G, Buchanan JA, Walker S, Marshall CR, Uddin M, Zarrei M, Deneault E, D’Abate L, Chan AJS, Koyanagi S, Paton T, Pereira SL, Hoang N, Engchuan W, Higginbotham EJ, Ho K, Lamoureux S, Li W, MacDonald JR, Nalpathamkalam T, Sung WWL, Tsoi FJ, Wei J, Xu L, Tasse A-M, Kirby E, Van Etten W, Twigger S, Roberts W, Drmic I, Jilderda S, Modi BM, Kellam B, Szego M, Cytrynbaum C, Weksberg R, Zwaigenbaum L, Woodbury-Smith M, Brian J, Senman L, Iaboni A, Doyle-Thomas K, Thompson A, Chrysler C, Leef J, Savion-Lemieux T, Smith IM, Liu X, Nicolson R, Seifer V, Fedele A, Cook EH, Dager S, Estes A, Gallagher L, Malow BA, Parr JR, Spence SJ, Vorstman J, Frey BJ, Robinson JT, Strug LJ, Fernandez BA, Elsabbagh M, Carter MT, Hallmayer J, Knoppers BM, Anagnostou E, Szatmari P, Ring RH, Glazer D, Pletcher MT, Scherer SW. 2017. Whole genome sequencing resource identifies 18 new candidate genes for autism spectrum disorder. *Nat Neurosci* **20**:602–611. doi:10.1038/nn.4524

Fiorillo C, Moro F, Yi J, Weil S, Brisca G, Astrea G, Severino M, Romano A, Battini R, Rossi A, Minetti C, Bruno C, Santorelli FM, Vallee R. 2014. Novel dynein DYNC1H1 neck and motor domain mutations link distal spinal muscular atrophy and abnormal cortical development. *Hum Mutat* **35**:298–302. doi:10.1002/humu.22491

González-Morón D, Vishnopolska S, Consalvo D, Medina N, Marti M, Córdoba M, Vazquez-Dusefante C, Claverie S, Rodríguez-Quiroga SA, Vega P, Silva W, Kochen S, Kauffman MA. 2017. Germline and somatic mutations in cortical malformations: Molecular defects in Argentinean patients with neuronal migration disorders. *PLoS One* **12**:e0185103. doi:10.1371/journal.pone.0185103

Hayashi S, Uehara DT, Tanimoto K, Mizuno S, Chinen Y, Fukumura S, Takanashi J-I, Osaka H, Okamoto N, Inazawa J. 2017. Comprehensive investigation of CASK mutations and other genetic etiologies in 41 patients with intellectual disability and microcephaly with pontine and cerebellar hypoplasia (MICPCH). *PLoS One* **12**:e0181791. doi:10.1371/journal.pone.0181791

Iossifov I, O’Roak BJ, Sanders SJ, Ronemus M, Krumm N, Levy D, Stessman HA, Witherspoon KT, Vives L, Patterson KE, Smith JD, Paeper B, Nickerson DA, Dea J, Dong S, Gonzalez LE, Mandell JD, Mane SM, Murtha MT, Sullivan CA, Walker MF, Waqar Z, Wei L, Willsey AJ, Yamrom B, Lee Y, Grabowska E, Dalkic E, Wang Z, Marks S, Andrews P, Leotta A, Kendall J, Hakker I, Rosenbaum J, Ma B, Rodgers L, Troge J, Narzisi G, Yoon S, Schatz MC, Ye K, McCombie WR, Shendure J, Eichler EE, State MW, Wigler M. 2014. The contribution of de novo coding mutations to autism spectrum disorder. *Nature* **515**:216–221. doi:10.1038/nature13908

Jamuar SS, Lam A-TN, Kircher M, D’Gama AM, Wang J, Barry BJ, Zhang X, Hill RS, Partlow JN, Rozzo A, Servattalab S, Mehta BK, Topcu M, Amrom D, Andermann E, Dan B, Parrini E, Guerrini R, Scheffer IE, Berkovic SF, Leventer RJ, Shen Y, Wu BL, Barkovich AJ, Sahin M, Chang BS, Bamshad M, Nickerson DA, Shendure J, Poduri A, Yu TW, Walsh CA. 2014. Somatic mutations in cerebral cortical malformations. *The New England journal of medicine* **371**:733–743. doi:10.1056/NEJMoa1314432

Laquerriere A, Maillard C, Cavallin M, Chapon F, Marguet F, Molin A, Sigaudy S, Blouet M, Benoist G, Fernandez C, Poirier K, Chelly J, Thomas S, Bahi-Buisson N. 2017. Neuropathological Hallmarks of Brain Malformations in Extreme Phenotypes Related to DYNC1H1 Mutations. *J Neuropathol Exp Neurol* **76**:195–205. doi:10.1093/jnen/nlw124

Meng L, Pammi M, Saronwala A, Magoulas P, Ghazi AR, Vetrini F, Zhang J, He W, Dharmadhikari AV, Qu C, Ward P, Braxton A, Narayanan S, Ge X, Tokita MJ, Santiago-Sim T, Dai H, Chiang T, Smith H, Azamian MS, Robak L, Bostwick BL, Schaaf CP, Potocki L, Scaglia F, Bacino CA, Hanchard NA, Wangler MF, Scott D, Brown C, Hu J, Belmont JW, Burrage LC, Graham BH, Sutton VR, Craigen WJ, Plon SE, Lupski JR, Beaudet AL, Gibbs RA, Muzny DM, Miller MJ, Wang X, Leduc MS, Xiao R, Liu P, Shaw C, Walkiewicz M, Bi W, Xia F, Lee B, Eng CM, Yang Y, Lalani SR. 2017. Use of Exome Sequencing for Infants in Intensive Care Units: Ascertainment of Severe Single-Gene Disorders and Effect on Medical Management. *JAMA Pediatr* **171**:e173438. doi:10.1001/jamapediatrics.2017.3438

Mineyko A, Doja A, Hurteau J, Dobyns WB, Das S, Boycott KM. 2010. A novel missense mutation in LIS1 in a child with subcortical band heterotopia and pachygyria inherited from his mildly affected mother with somatic mosaicism. *J Child Neurol* **25**:738–741. doi:10.1177/0883073809343312

Poirier K, Lebrun N, Broix L, Tian G, Saillour Y, Boscheron C, Parrini E, Valence S, Pierre BS, Oger M, Lacombe D, Geneviève D, Fontana E, Darra F, Cances C, Barth M, Bonneau D, Bernadina BD, N’Guyen S, Gitiaux C, Parent P, des Portes V, Pedespan JM, Legrez V, Castelnau-Ptakine L, Nitschke P, Hieu T, Masson C, Zelenika D, Andrieux A, Francis F, Guerrini R, Cowan NJ, Bahi-Buisson N, Chelly J. 2013. Mutations in TUBG1, DYNC1H1, KIF5C and KIF2A cause malformations of cortical development and microcephaly. *Nature genetics* **45**:639–647. doi:10.1038/ng.2613

Saillour Y, Carion N, Quelin C, Leger P-L, Boddaert N, Elie C, Toutain A, Mercier S, Barthez MA, Milh M, Joriot S, des Portes V, Philip N, Broglin D, Roubertie A, Pitelet G, Moutard ML, Pinard JM, Cances C, Kaminska A, Chelly J, Beldjord C, Bahi-Buisson N. 2009. LIS1-related isolated lissencephaly: spectrum of mutations and relationships with malformation severity. *Arch Neurol* **66**:1007–1015. doi:10.1001/archneurol.2009.149

Scoto M, Rossor AM, Harms MB, Cirak S, Calissano M, Robb S, Manzur AY, Martínez Arroyo A, Rodriguez Sanz A, Mansour S, Fallon P, Hadjikoumi I, Klein A, Yang M, De Visser M, Overweg-Plandsoen WCGT, Baas F, Taylor JP, Benatar M, Connolly AM, Al-Lozi MT, Nixon J, de Goede CGEL, Foley AR, Mcwilliam C, Pitt M, Sewry C, Phadke R, Hafezparast M, Chong WKK, Mercuri E, Baloh RH, Reilly MM, Muntoni F. 2015. Novel mutations expand the clinical spectrum of DYNC1H1-associated spinal muscular atrophy. *Neurology* **84**:668–679. doi:10.1212/WNL.0000000000001269

Torres FR, Montenegro MA, Marques-De-Faria AP, Guerreiro MM, Cendes F, Lopes-Cendes I. 2004. Mutation screening in a cohort of patients with lissencephaly and subcortical band heterotopia. *Neurology* **62**:799–802. doi:10.1212/01.wnl.0000113725.46254.fd

Trujillano D, Bertoli-Avella AM, Kumar Kandaswamy K, Weiss ME, Köster J, Marais A, Paknia O, Schröder R, Garcia-Aznar JM, Werber M, Brandau O, Calvo Del Castillo M, Baldi C, Wessel K, Kishore S, Nahavandi N, Eyaid W, Al Rifai MT, Al-Rumayyan A, Al-Twaijri W, Alothaim A, Alhashem A, Al-Sannaa N, Al-Balwi M, Alfadhel M, Rolfs A, Abou Jamra R. 2017. Clinical exome sequencing: results from 2819 samples reflecting 1000 families. *Eur J Hum Genet* **25**:176–182. doi:10.1038/ejhg.2016.146

Uyanik G, Morris-Rosendahl DJ, Stiegler J, Klapecki J, Gross C, Berman Y, Martin P, Dey L, Spranger S, Korenke GC, Schreyer I, Hertzberg C, Neumann TE, Burkart P, Spaich C, Meng M, Holthausen H, Adès L, Seidel J, Mangold E, Buyse G, Meinecke P, Schara U, Zeschnigk C, Muller D, Helland G, Schulze B, Wright ML, Kortge-Jung S, Hehr A, Bogdahn U, Schuierer G, Kohlhase J, Aigner L, Wolff G, Hehr U, Winkler J. 2007. Location and type of mutation in the LIS1 gene do not predict phenotypic severity. *Neurology* **69**:442–447. doi:10.1212/01.wnl.0000266629.98503.d0

Vissers LELM, de Ligt J, Gilissen C, Janssen I, Steehouwer M, de Vries P, van Lier B, Arts P, Wieskamp N, del Rosario M, van Bon BWM, Hoischen A, de Vries BBA, Brunner HG, Veltman JA. 2010. A de novo paradigm for mental retardation. *Nat Genet* **42**:1109–1112. doi:10.1038/ng.712

Zhu X, Petrovski S, Xie P, Ruzzo EK, Lu Y-F, McSweeney KM, Ben-Zeev B, Nissenkorn A, Anikster Y, Oz-Levi D, Dhindsa RS, Hitomi Y, Schoch K, Spillmann RC, Heimer G, Marek-Yagel D, Tzadok M, Han Y, Worley G, Goldstein J, Jiang Y-H, Lancet D, Pras E, Shashi V, McHale D, Need AC, Goldstein DB. 2015. Whole-exome sequencing in undiagnosed genetic diseases: interpreting 119 trios. *Genet Med* **17**:774–781. doi:10.1038/gim.2014.191

Zillhardt JL, Poirier K, Broix L, Lebrun N, Elmorjani A, Martinovic J, Saillour Y, Muraca G, Nectoux J, Bessieres B, Fallet-Bianco C, Lyonnet S, Dulac O, Odent S, Rejeb I, Ben Jemaa L, Rivier F, Pinson L, Geneviève D, Musizzano Y, Bigi N, Leboucq N, Giuliano F, Philip N, Vilain C, Van Bogaert P, Maurey H, Beldjord C, Artiguenave F, Boland A, Olaso R, Masson C, Nitschké P, Deleuze J-F, Bahi-Buisson N, Chelly J. 2016. Mosaic parental germline mutations causing recurrent forms of malformations of cortical development. *Eur J Hum Genet* **24**:611–614. doi:10.1038/ejhg.2015.192
